# Pharmacodynamic change in plasma angiogenic proteins: a dose-escalation phase 1 study of the multi-kinase inhibitor lenvatinib

**DOI:** 10.1186/1471-2407-14-530

**Published:** 2014-07-21

**Authors:** Noriyuki Koyama, Kenichi Saito, Yuki Nishioka, Wataru Yusa, Noboru Yamamoto, Yasuhide Yamada, Hiroshi Nokihara, Fumiaki Koizumi, Kazuto Nishio, Tomohide Tamura

**Affiliations:** 1 Oncology Medical Department, Eisai Co, Ltd, Tokyo, Japan; 2 Japan Biostatistics/Biostatistics/Clinical Science, Scientific and Operational Clinical Support Core Function Unit, Eisai Co, Ltd, Tokyo, Japan; 3 Japan Clinical Pharmacology/Clinical Pharmacology/Clinical Science, Scientific and Operation Clinical Support Core Function Unit, Eisai Co, Ltd, Tokyo, Japan; 4 Oncology Clinical Development Section, Japan/Asia Clinical Research Production Creation Unit, Eisai Co, Ltd, Tokyo, Japan; 5 Department of Thoracic Oncology, National Cancer Center Hospital, Tokyo, Japan; 6 Department of Gastrointestinal Oncology, National Cancer Center Hospital, Tokyo, Japan; 7 Shien-Lab and Support Facility of Project Ward, National Cancer Center Hospital, Tokyo, Japan; 8 Department of Genome Biology, Kinki University School of Medicine, Osaka, Japan

**Keywords:** Lenvatinib, Angiogenesis, Pharmacodynamic biomarkers, VEGF, SDF1α, sVEGFR2, Maximum tumor shrinkage

## Abstract

**Background:**

Lenvatinib (E7080), an oral multi-kinase inhibitor, has inhibitory action on tumor cell proliferation and tumor angiogenesis in preclinical models. We evaluated correlations between pharmacodynamic (PD) biomarkers with patient clinical outcomes in a lenvatinib phase 1 dose-escalation study.

**Methods:**

Plasma angiogenic proteins were evaluated as potential PD biomarkers of response to lenvatinib in a dose-escalation phase 1 study. Lenvatinib was administered to 27 patients by twice-daily dosing in 3-week cycles; 2 weeks of treatment followed by 1 week of rest until discontinuation. Blood samples for plasma proteins were collected on days 1 (baseline), 8, and 15 of cycle 1, and days 1, 8, and 15 of cycle 2. Selected clinical outcomes, including tumor shrinkage and adverse events (AEs), were used for correlative analyses of pharmacokinetic parameters and PD biomarkers.

**Results:**

Tumor shrinkage and changes in PD biomarkers (increased vascular endothelial growth factor [VEGF] and stromal cell-derived factor 1 alpha [SDF1α] levels and decreased soluble VEGF receptor 2 [sVEGFR2] levels) significantly correlated with increasing lenvatinib exposure. Observed changes in levels of VEGF, SDF1α, and sVEGFR2 were maintained on day 15 of cycle 1, but returned to baseline during the 1-week rest period, and similar changes were induced by reinstitution of treatment in cycle 2. The worst grades of hypertension, proteinuria, and fatigue were associated with changes in VEGF and HGF at day 8 of cycle 1. Maximum tumor shrinkage was correlated with increased SDF1α levels. Decreased sVEGFR2 level was also correlated with tumor shrinkage and frequency of hypertension, proteinuria, and fatigue. Tumor shrinkage significantly correlated with the worst grade of proteinuria, but not with hypertension or fatigue.

**Conclusion:**

PD biomarker changes observed in plasma angiogenic proteins are correlated with lenvatinib-induced tumor shrinkage and AEs. Our findings warrant further assessment of plasma proteins associated with angiogenesis as potential biomarkers of lenvatinib activity.

**Trial registration:**

ClinicalTrial.gov: NCT00280397 (January 20, 2006).

## Background

Various agents that inhibit tumor angiogenesis have recently been approved or are currently being developed in clinical trials [1-4]. Although treatment benefits are often seen early during the course of antiangiogenic therapy, therapy is often discontinued when tumors develop resistance and resume growth. Additionally, accumulation of biologic changes in host tissue may result in unacceptable toxicities that necessitate dose interruptions or reductions, resulting in decreased dose density and potentially lower efficacy.

Compensatory mechanisms for resistance may be acquired by the tumor and host tissues as a response to vascular damage and elevated tumor hypoxia, and include upregulation of alternative proangiogenic factors. A recent study indicated that stable microvasculature kept disseminated tumor cells dormant, whereas sprouting neovasculature sparked micrometastatic outgrowth [5]. Proangiogenic factors derived from tumor tissues include platelet-derived growth factor (PDGF), placental growth factor (PlGF), basic fibroblast growth factor (bFGF), and stromal cell-derived factor1 alpha (SDF1α). Stromal cells surrounding a tumor, such as tumor-associated fibroblasts, can upregulate PDGF-C and activate pericytes, which also play a role in maintaining vascular integrity and developing resistance in response to inhibition of vascular endothelial growth factor (VEGF) [6]. In addition, a variety of bone-marrow-derived cells may mediate resistance to VEGF inhibition by producing proangiogenic factors [7,8]. Some tumors develop resistance to VEGF inhibitors by secreting cytokines that recruit myeloid cells and other cells that promote angiogenesis and immune tolerance, thereby affecting the efficacy and safety of anti-VEGF therapy [9].

The development of biomarkers of clinical efficacy and safety may provide important clinical insight for the appropriate selection of patients and management of antiangiogenesis therapy. Early prediction of efficacy and toxicity with plasma biomarkers related to angiogenesis may contribute to optimal patient care. In addition, potential insight into the mechanisms of resistance may lead to the development of rational combinations of antiangiogenic treatment with agents that inhibit other signaling pathways that promote resistance to antiangiogenic therapy [1,10].

Over the past decade, a multiplex protein assay has been validated that enables identification of multiple changes in the levels of plasma proteins in preclinical and clinical samples. In preclinical studies, treatment with the VEGF receptor (VEGFR) inhibitor sunitinib induced dose-dependent increases in VEGF and PlGF levels and decreases in soluble VEGFR 2 (sVEGFR2) levels, while treatment with cetuximab, an epidermal growth factor receptor antibody, increased transforming growth factor alpha levels in a tumor-independent manner [11,12]. These data suggest that changes in the levels of plasma proteins may reflect the biologic response of host tissues to therapy and may be useful markers for the clinical activity of antitumor agents.

Lenvatinib (E7080) is an oral multiple tyrosine kinase inhibitor (TKI) of VEGFR1–3, fibroblast growth factor receptor 1–4, PDGF receptor alpha (-α), RET protein, and c-Kit protein. Inhibition of xenograft tumor growth by lenvatinib was observed at doses as low as 1.0 and 10.0 mg/kg [13-15]. In phase 1 and 2 clinical trials, lenvatinib demonstrated antitumor activity and a manageable toxicity profile as a single agent [16-18]. In a phase 1 dose-escalation study, lenvatinib showed preliminary activity for durable disease control in a variety of tumor types, including a partial response in a patient with colon cancer and stable disease in 84% of evaluable patients [17]. Lenvatinib has a manageable toxicity profile with adverse events (AEs) consistent with other anti-VEGF treatments, including hypertension, proteinuria, and fatigue [16,17,19]. In this phase 1 dose-escalation study, we analyzed the pharmacodynamic (PD) changes in angiogenic plasma proteins during cycles 1 and 2 of lenvatinib treatment.

## Methods

### Study design

This single-center, open-label, sequential dose-escalation study of lenvatinib was conducted at the National Cancer Center Hospital, Tokyo, Japan. Lenvatinib was orally administered twice daily in 3-week cycles (2 weeks on/1 week off) in patients with advanced solid tumors. Pharmacokinetic (PK) parameters, safety, tolerability, efficacy, and exploratory PD markers were examined. Eligible patients were sequentially enrolled on escalating doses of oral lenvatinib with a standard 3 + 3 design. AEs were monitored throughout the treatment cycles. Best tumor response and disease progression were measured using the Response Evaluation Criteria in Solid Tumors (RECIST), version 1.0 [20]. Tumors were assessed at screening, in cycle 2 or 3, and in every 2 cycles thereafter. This study was performed in accordance with the ethical principles stipulated by the Declaration of Helsinki and Good Clinical Practice guidelines, and approved by the Institutional Review Board at the National Cancer Center Hospital, Tokyo, Japan. All patients provided written, informed consent before screening.

### Pharmacokinetic and pharmacodynamic analyses

Blood samples for PK and PD analyses were collected from each patient. Plasma lenvatinib concentrations were determined with liquid chromatography/tandem mass spectrometry by Sumitomo Chemical Co. Ltd (Osaka, Japan). Area under the curve (AUC) was calculated from the data obtained at steady state in cycle 1. Plasma proteins were measured with a BioPlex assay (Bio-Rad Laboratories, Inc) by Mitsubishi Chemical Medience Corp (Ibaraki, Japan). Plasma PD biomarkers measured in this study included: interleukin (IL)-6, IL-8, and IL-10; VEGF; PDGF; hepatocyte growth factor (HGF); stem cell factor (SCF); and SDF1α. sVEGFR1 and sVEGFR2 were measured by enzyme-linked immunosorbent assay.

### Statistical analysis

PK parameters of plasma lenvatinib concentration-vs-time data were examined by noncompartmental analysis using WinNonlin version 5.2 software (Pharsight Corporation, Mountain View, CA, USA). Correlation analyses between PK, PD, and clinical outcomes were performed using Spearman’s rank correlation coefficient, and Wilcoxon signed rank test was used to determine change from pretreatment. Multiplicity adjustments were not conducted. Maximum tumor shrinkage (%) was defined as the percentage of change from baseline in the sum of tumor diameters of target lesions at the maximum shrinkage observed.

## Results

**Figure 1:**
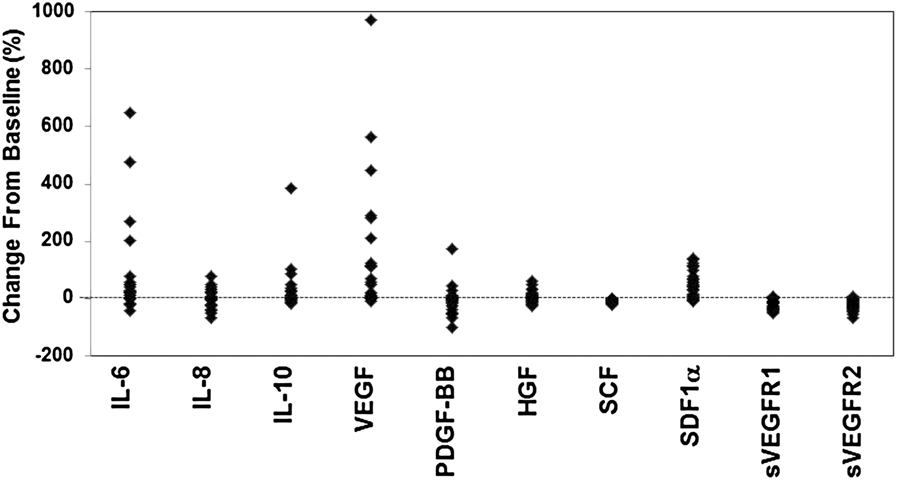
**Changes in plasma proteins after lenvatinib treatment.** The concentrations of plasma proteins were measured at baseline and at day 8 of lenvatinib treatment in individual patients, and the percentage change from baseline was plotted for each patient.

Twenty-seven patients were enrolled in the study. Because change in plasma proteins is hypothesized to reflect biologic response to treatment and may be a marker of clinical activity, we examined whether lenvatinib treatment altered the levels of putative PD biomarkers (Figure 1). We measured a total of 20 plasma angiogenic proteins and cytokines at baseline and after treatment [17], and found that levels of IL-6, IL-10, VEGF, HGF, and SDF1α were increased, whereas levels of PDGF-BB, sVEGFR1, and sVEGFR2 were decreased at day 8 of lenvatinib treatment. IL-8 and SCF levels were increased in some patients but decreased in others.

**Figure 2:**
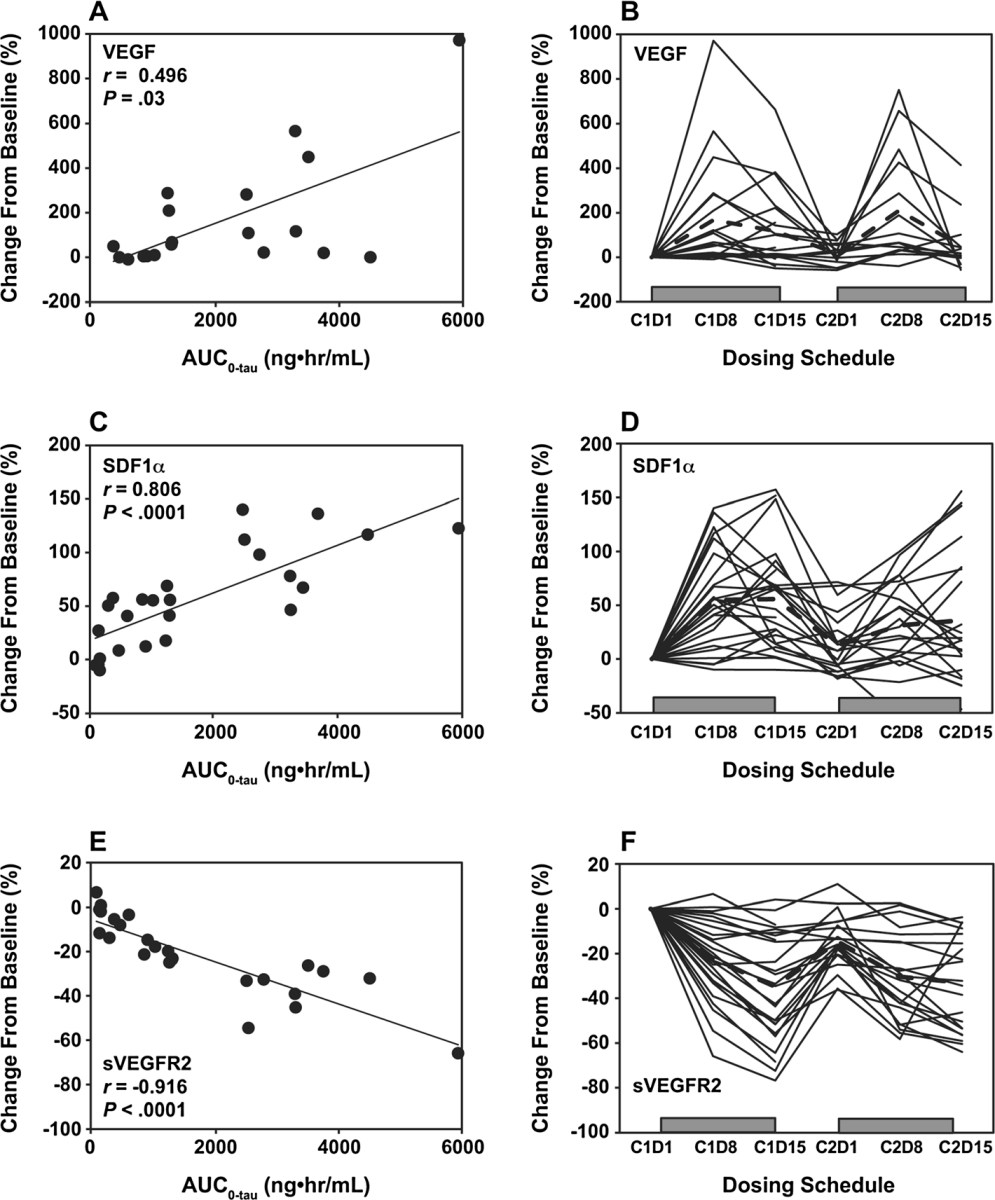
**Lenvatinib treatment-dependent changes in VEGF, SDF1α, and sVEGFR2.** The concentrations of plasma VEGF **(A)**, SDF1α **(C)**, and sVEGFR2 **(E)** were measured at baseline and at day 8 of lenvatinib treatment, and the percentage change from baseline was plotted in correlation with AUC_0-tau_. The correlation coefficient (*r*) and *P* value in each analysis are indicated. The percentage PD changes in VEGF **(B)**, SDF1α **(D)**, and sVEGFR2 **(F)** relative to dosing schedule were indicated for 14 days on treatment (at days [D] 8 and 15 of cycle [C] 1), after 7 days off treatment, and on retreatment in cycle 2. A dotted line indicates the mean percentage of change, and gray boxes indicate each on-treatment period.

We next investigated AUC-dependent changes in PD biomarker levels in plasma proteins and correlations with area under the curve for the dosing interval (AUC_0-tau_; Table 1). Only the increased levels of VEGF and SDF1α and the decreased level of sVEGFR2 were significantly correlated with AUC_0-tau_. Correlation coefficients and *P* values, respectively, were 0.496 and .030 for VEGF, 0.806 and < .0001 for SDF1α, and -0.916 and < .0001 for sVEGFR2. Similar correlations were seen in the analysis with maximum and minimum concentrations (data not shown). Relative to the dosing schedule, PD changes in these proteins were induced on day 8 of cycle 1 and maintained on day 15 of cycle 1, but returned to baseline during the 1-week rest period. Similar changes were induced by reinstitution of treatment in cycle 2, suggesting that these PD biomarker changes were associated with lenvatinib treatment (Figure 2).

**Table 1:** **Correlation between lenvatinib treatment**-**dependent changes in plasma biomarkers and AUC**

**Plasma Biomarker**	**n**	**Correlation With AUC**_**0**-**tau**_	
		***r***	***P *** **Value**
IL-6	19	-0.100	.683
IL-8	19	-0.202	.407
IL-10	19	0.061	.802
VEGF	19	0.496	.030
PDGF-BB	19	-0.161	.509
HGF	25	0.263	.203
SCF	25	-0.210	.313
SDF1α	25	0.806	< .0001
sVEGFR1	25	-0.378	.062
sVEGFR2	25	-0.916	< .0001

The concentrations of plasma proteins were measured at baseline and at day 8 of lenvatinib treatment, and the percentage change from baseline was analyzed in correlation with AUC_0-tau_. Spearman’s correlation coefficient (*r*) and *P* value (p) for each analysis is listed.

**Figure 3:**
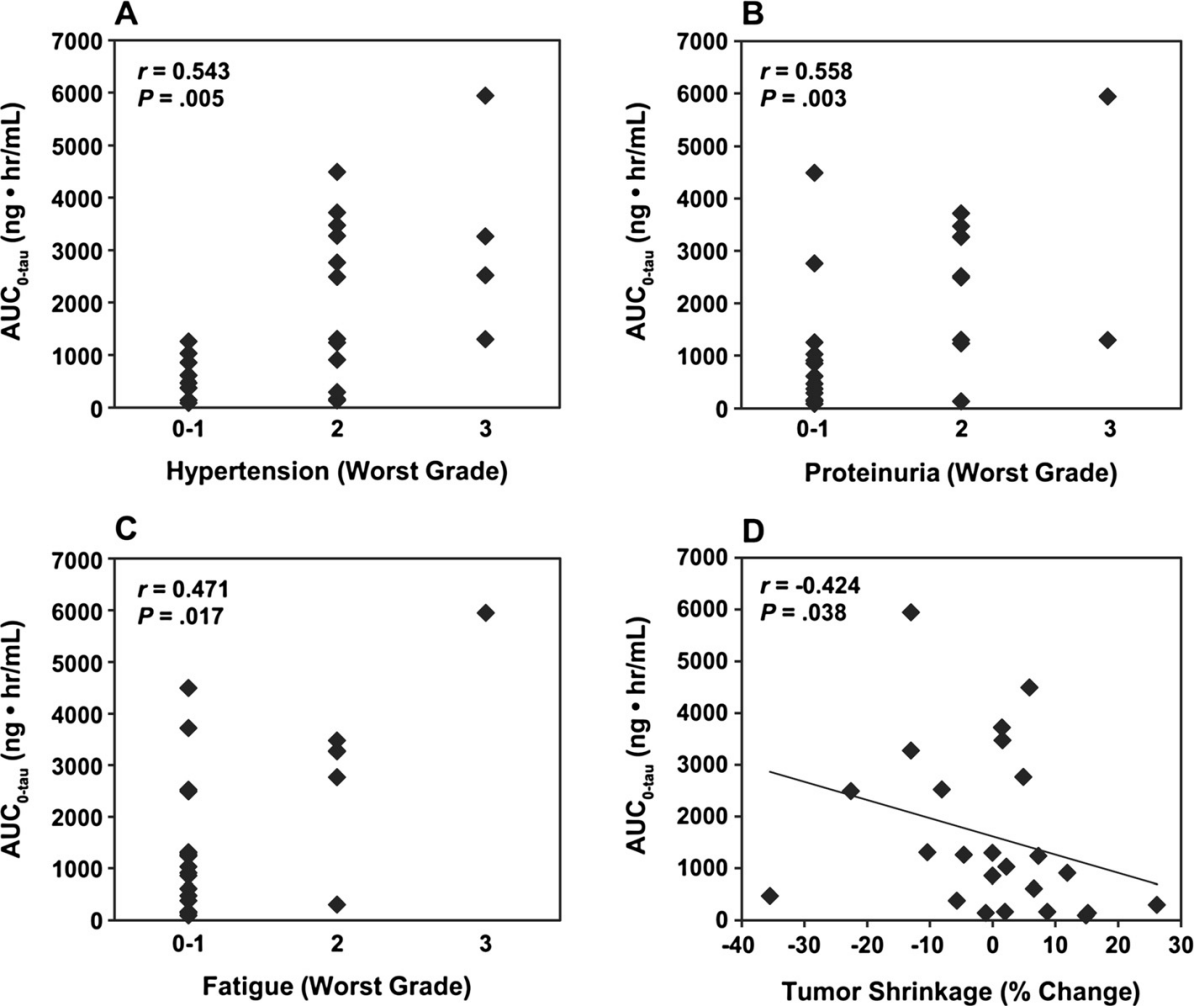
**Spearman’s correlation analysis of AUC with toxicity and tumor shrinkage induced by lenvatinib.** The worst grade of hypertension **(A)**, proteinuria **(B)**, and fatigue **(C)** and the maximum tumor shrinkage **(D)** for the treatment duration were analyzed in correlation with AUC_0-tau_. The correlation coefficient (*r*) and *P* value for each analysis is indicated.

Correlation analyses of AEs and tumor shrinkage with AUC_0-tau_ were also performed. In a previous study, the most frequent AEs associated with lenvatinib treatment were hypertension, proteinuria, and fatigue [17]. Using the worst grade of each of these AEs over the duration of treatment in correlation with AUC_0-tau_, Spearman’s rank correlation analysis indicated significant correlation of hypertension (*P* = .005), proteinuria (*P* = .003), and fatigue (*P* = .017) with AUC_0-tau_ (Figure 3A-C). Correlation analyses of other AEs were not performed, because other AEs occurred in a limited number of patients [17]. The analysis of maximum tumor shrinkage and AUC_0-tau_ yielded a significant but weak correlation (*P* = .038; Figure 3D). The results of correlation analysis of toxicities and tumor shrinkage with the PD change in plasma proteins at cycle 1 are listed in Table 2. The analysis showed a significant correlation between change in VEGF and HGF levels in cycle 1 with the worst grades of hypertension, proteinuria, and fatigue. Additionally, maximum tumor shrinkage showed a significant correlation with PD change in SDF1α levels, where patients with a greater increase in SDF1α levels had greater tumor shrinkage. However, no correlations with tumor shrinkage were seen for VEGF or HGF. Decreased sVEGFR2 level was also correlated with tumor shrinkage and frequency of hypertension, proteinuria, and fatigue.

**Table 2:** Correlation of toxicities and tumor shrinkage with percentage change in plasma biomarkers

**Plasma Biomarker**	**n**	**Hypertension**		**Proteinuria**		**Fatigue**		**Tumor Shrinkage**	
		***r***	***P *** **Value**	***r***	***P *** **Value**	***r***	***P *** **Value**	***r***	***P *** **Value**
IL-6	19	0.19	.421	0.247	.294	-0.173	.465	-0.437	.061
IL-8	19	-0.077	.748	0.053	.823	0.002	.993	-0.191	.434
IL-10	19	0.158	.505	0.038	.872	0.141	.552	-0.392	.097
VEGF	19	0.569	.008^b^	0.703	<.001^a^	0.529	.016^c^	-0.277	.250
PDGF-BB	19	-0.215	.363	-0.151	.524	0.043	.857	-0.277	.250
HGF	25	0.624	<.001^a^	0.615	<.001^a^	0.431	.027^c^	-0.235	.257
SCF	25	0.145	.478	0.176	.390	-0.057	.780	0.261	.207
SDF1α	25	0.257	.204	0.314	.117	0.344	.085	0.424	.034^c^
sVEGFR1	25	-0.38	.055	-0.365	.066	0.065	.753	0.038	.855
sVEGFR2	25	-0.613	<.001^a^	-0.601	.001^b^	-0.466	.016^c^	-0.431	.031^c^

The concentrations of plasma proteins were measured at baseline and at day 8 of lenvatinib treatment, and the percentage change from baseline was analyzed (Spearman’s correlation analysis) in correlation with toxicities and tumor shrinkage. Worst grade of toxicities and maximum tumor shrinkage over the duration of treatment were used for the analysis.

^a^*P* < 0.001.

^b^*P* < 0.01.

^c^*P* < 0.05.

**Figure 4:**
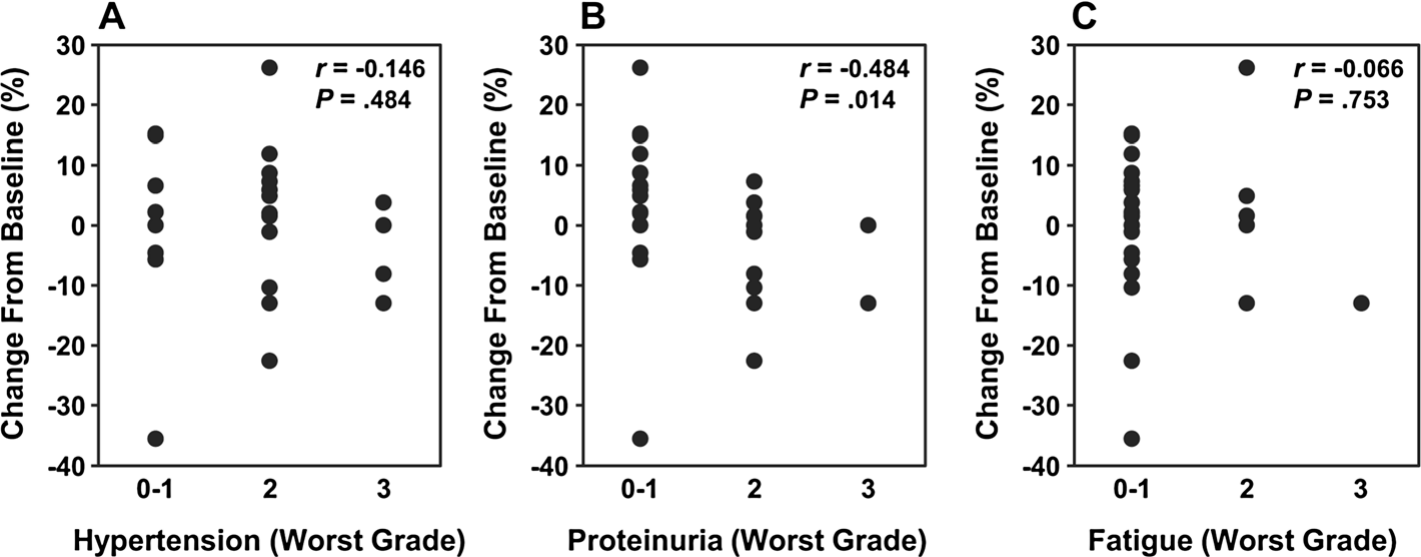
**Correlation of tumor shrinkage with the worst grade of toxicity.** Correlation analyses were performed for maximum tumor shrinkage percentage change from baseline and the worst grade of hypertension **(A)**, proteinuria **(B)**, and fatigue **(C)** over the treatment duration. The correlation coefficient (*r*) and *P* value for each analysis is indicated.

Finally, a correlation analysis of AEs with maximum tumor shrinkage is shown in Figure 4. Although tumor shrinkage and worst grade in hypertension, proteinuria, and fatigue were significantly correlated with AUC_0-tau_ (Figure 3), a significant correlation between tumor shrinkage and worst grade of AE was only observed for proteinuria (*P* = .014; Figure 4B).

## Discussion

In this study, we have observed significant correlations of toxicity and tumor shrinkage with PK parameters and PD changes in VEGF, SDF1α, and sVEGFR2 levels. While evaluating PK parameters requires multiple samplings and analyses, PD changes in plasma markers are more easily monitored. More importantly, PD biomarkers may reflect biologic changes in tumor and host tissues in response to treatment and are potentially useful for patient monitoring.

An adaptive treatment approach based on the incidence of toxicity may be effective in maintaining treatment and increasing treatment benefits of VEGF inhibitors [19]. The development of both treatment-related hypertension and proteinuria has been reported in patients receiving lenvatinib therapy [17,19], as well as in clinical studies of other inhibitors of the VEGF signaling pathway [21,22]. We have observed that changes in the levels of VEGF and HGF in cycle 1 correlated with the worst grade of hypertension, proteinuria, and fatigue. Monitoring plasma levels of VEGF and HGF may help predict toxicity, and by identifying those patients who require increased surveillance, it may lessen the risk of AE incidence or worsening severity.

The effects of VEGF and HGF on blood pressure may be explained by their induction of endothelial proliferation and contribution to the protection and repair of vascular endothelial cells [23]. HGF may be upregulated in response to elevated blood pressure to counter endothelial dysfunction. This concept is supported by recent reports that HGF treatment produced therapeutic benefit against peripheral arterial disease [24,25].

The relationship between increased levels of VEGF and HGF with fatigue, however, is not clear. Elevated VEGF was significantly associated with increased fatigue in anthracycline-based chemotherapy in breast cancer [26]. Additionally, correlations were reported between lower serum HGF levels and fatigue in healthy control participants, as well as between increased serum HGF levels and antidepressant efficacy in patients with panic disorder [27]. Hypothyroidism has been reported in sorafenib-treated patients with renal cancer and sunitinib-treated patients with gastrointestinal stromal cancer [28,29], and anti-VEGF or anti-VEGFR2 treatment induced vascular regression in the thyroid and decreased plasma thyroid hormone levels in mice [30]. Additionally, thyroid hormone replacement therapy improved fatigue in axitinib-treated patients with cancer [31]. These reports support the need for further biomarker analyses to elucidate the role of VEGF and HGF in thyroid function.

Increased SDF1α levels were also correlated with greater tumor shrinkage. Activation of the immune pathway is important for tumor shrinkage; SDF1α and its receptor CXCR4 play important roles in immune function and have the potential to enhance anticancer immunity [32,33]. SDF1α and CXCR4 also enhance progenitor cell accumulation at angiogenic sites and are important biomarkers of antiangiogenic therapy resistance [34]. We have previously reported that higher baseline SDF1α levels correlated with shorter treatment duration [17]. The role of SDF1α as a potential PD biomarker of resistance to lenvatinib treatment needs further study, especially because baseline and subsequent changes from baseline levels of SDF1α may be interpreted differently.

VEGFR2 is one of the most important mediators of angiogenesis in normal and tumor tissues [35]. We have observed decreases in levels of sVEGFR1 and sVEGFR2 after lenvatinib treatment, while decreased sVEGFR2 levels were correlated with PK parameters, AE frequency, and tumor shrinkage. Soluble forms of VEGFR1 and VEGFR2 are induced through alternative splicing of VEGFR1 and VEGFR2 transcripts and act as inhibitors of VEGF signaling [36,37]. TKI treatment-associated decreases in circulating sVEGFR2 levels have been consistently observed [38-40], but their clinical relevance remains controversial. A possible interpretation is that a decreased level of sVEGFR2 is a surrogate index for PK parameters such as AUC, which was correlated with both AEs and tumor shrinkage in our phase 1 study. A study of axitinib in renal cell carcinoma indicated that patients with greater decreases in sVEGFR2 levels showed higher objective response rates and longer progression-free survival (PFS) than those with smaller decreases [41]. Recent results of a trial evaluating cediranib in hepatocellular carcinoma found that PFS was inversely correlated with baseline levels of sVEGFR2 [38]. Alternatively, higher levels of sVEGFR1 and lower levels of sVEGFR2 were related to organ dysfunction in patients with disseminated intravascular coagulation [42]. The role of sVEGFR2 as a biomarker remains is not yet understood, and further analysis will be necessary to examine its potential as a predictive biomarker of lenvatinib activity.

Predictive plasma biomarkers of survival, including PFS and overall survival (OS), may greatly inform patient care and management. Higher baseline VEGF levels in plasma were correlated with shorter OS in sorafenib-treated patients with renal cancer and hepatocellular carcinoma [43,44]. Higher baseline levels of VEGF and IL-8 were associated with shorter PFS and OS in sunitinib-treated patients with renal cancer [45]. PFS and OS were not analyzed in this dose-escalation phase I study enrolling patients with various tumor types and treatment history; therefore correlation analyses with survival was not performed. However, our previous report indicated the inverse correlation of lenvatinib treatment duration with baseline levels of SDF1α, but not VEGF or IL-8 [17]. Potential predictive biomarkers of PFS and OS for lenvatinib are under investigation in ongoing phase 2 and 3 studies of lenvatinib.

Hypertension and proteinuria are major toxicities of antiangiogenic VEGF inhibitors, and their onset may suggest inhibition of the VEGF/VEGFR pathway. However, the hypothesis that hypertension and proteinuria are biomarkers of response to antiangiogenic drugs remains inconclusive [46]. Differences in the definition of toxicity used for correlation analysis and in the study criteria of baseline disease, as well as use of concomitant agents, may affect the analysis. In this study, tumor shrinkage by lenvatinib was significantly correlated with proteinuria, but not with hypertension or fatigue. Because tumor shrinkage by antitumor agents is tumor type–specific, further analysis will be necessary in future phase 2 and 3 studies to examine the predictive value of toxicities for clinical efficacy of lenvatinib.

The PD change in plasma proteins may reflect a biologic response to lenvatinib treatment. In this study, PD biomarker changes were associated with lenvatinib treatment and were diminished during the 1-week rest period. These data suggest that the continuous administration of lenvatinib may maintain clinical activity. This continuous dosing regimen was adopted in subsequent lenvatinib studies [18,47].

## Conclusion

The analysis of lenvatinib-induced changes in the levels of plasma biomarkers related to angiogenesis suggested that angiogenesis inhibition may be correlated with clinical outcomes in patients with a wide range of solid tumors. Further study of the levels of angiogenic PD biomarkers and their potential relation to clinical outcomes with lenvatinib treatment in solid tumor types appears warranted and may inform treatment decisions.

## Abbreviations

AEs: Adverse events; AUC: Area under the curve; bFGF: Basic fibroblast growth factor; HGF: Hepatocyte growth factor; IL: Interleukin; OS: Overall survival; PD: Pharmacodynamic; PDGF: Platelet-derived growth factor; PDGFRα: PDGF receptor alpha; PFS: Progression-free survival; PK: Pharmacokinetic; PlGF: Placental growth factor; RECIST: Response Evaluation Criteria in Solid Tumors; SCF: Stem cell factor; SDF1α: Stromal cell-derived factor-1 alpha; TKI: Tyrosine kinase inhibitor; VEGF: Vascular endothelial growth factor; VEGFR: VEGF receptor; sVEGFR: Soluble VEGFR.

## Competing interests

NK, KS, YN, and WY are employees of Eisai Co Ltd. All other authors (NY, YY, HN, FK, KN, and TT) declare that they have no competing interests.

## Authors’ contributions

All of the authors have made substantial contributions to the conception and design of this study and the interpretation of data. NY, YY, HN, and TT participated in the acquisition of data in the clinical facility, and KS and YN performed the PK and PD analysis. NK, WY, and TT have been involved in drafting the manuscript and revising it critically for important intellectual content. TT gave final approval for the published version. All authors read and approved the final manuscript.

## Pre-publication history

The pre-publication history for this paper can be accessed here:

http://www.biomedcentral.com/1471-2407/14/530/prepub
